# Prevalence of anthelmintic resistance of gastrointestinal nematodes in Polish goat herds assessed by the larval development test

**DOI:** 10.1186/s12917-020-02721-9

**Published:** 2021-01-07

**Authors:** Marcin Mickiewicz, Michał Czopowicz, Agata Moroz, Adrian-Valentin Potărniche, Olga Szaluś-Jordanow, Marina Spinu, Paweł Górski, Iwona Markowska-Daniel, Marián Várady, Jarosław Kaba

**Affiliations:** 1 Division of Veterinary Epidemiology and Economics, Institute of Veterinary Medicine, Warsaw University of Life Sciences, Nowoursynowska 159c, 02-786 Warsaw, Poland; 2 Department of Infectious Diseases and Preventive Medicine, Law and Ethics, University of Agricultural Sciences and Veterinary Medicine, Calea Mănăștur 3-5, 400372 Cluj-Napoca, Romania; 3 Department of Small Animal Diseases with Clinic, Institute of Veterinary Medicine, Warsaw University of Life Sciences-SGGW, Nowoursynowska 159c, 02-786 Warsaw, Poland; 4 Division of Parasitology and Invasiology, Department of Preclinical Sciences, Institute of Veterinary Medicine, Warsaw University of Life Sciences-SGGW, Ciszewskiego 8, 02-786 Warsaw, Poland; 5 Institute of Parasitology, Slovak Academy of Sciences, Hlinkova 3, 04001 Košice, Slovakia

**Keywords:** Anthelmintic resistance, Gastrointestinal nematodes, Larval development test, Goats, benzimidazoles, Macrocyclic lactones, Levamisole

## Abstract

**Background:**

Helminthic infections, in particular those caused by gastrointestinal nematodes (GIN), are found worldwide and are among the most economically important diseases of goats. Anthelmintic resistance (AR) in GIN of goats is currently present worldwide, and single- or multidrug resistant species are widespread. The aim of this study was to determine the prevalence of AR to benzimidazoles (BZ), macrocyclic lactones (ML) and imidazothiazoles represented by levamisole (LEV) in the Polish goat herds by using an in vitro larval development test, which is useful especially in large-scale epidemiological surveys.

**Results:**

This cross-sectional study was conducted from September 2018 to June 2019 and enrolled 42 dairy goat herds scattered over the entire country. The most commonly used anthelmintic class in goat herds in Poland were BZ (92%), followed by ML (85%) and LEV (13%). BZ-resistant GIN populations were found in 37 herds (88%, CI 95%: 75 to 95%), ML-resistant GIN populations in 40 herds (95%, CI 95, 84 to 99%), and LEV-resistant GIN populations in 5 herds (12%, CI 95%: 5 to 25%). Multidrug resistance involving all three anthelmintic classes was found in 5 herds (12%, CI 95, 5 to 25%). Based on the morphological features of stage 3 larvae the main resistant GIN turned out to be *Haemonchus contortus* and *Trichostrongylus* spp. The use of BZ and frequency of anthelmintic treatments were significantly related to the presence of AR to BZ in Polish goat herds.

**Conclusions:**

This cross-sectional study demonstrates the existence of AR to BZ, ML and LEV on Polish goat farms. Resistance to BZ and ML is widespread, while AR to LEV is currently at a low level. A considerable proportion of herds harbours multidrug resistant GIN, which requires further consideration. An effective anthelmintic treatment strategy, reasonable preventive measures and better understanding of the resistance-related management practices by farmers and veterinarians may delay further development of AR.

**Supplementary Information:**

The online version contains supplementary material available at 10.1186/s12917-020-02721-9.

## Background

Parasitic infections, especially those caused by gastrointestinal nematodes (GIN), are one of the main factors responsible for economic losses in goat farming around the world [1]. Their control is mainly based on the use of three chemical classes of anthelmintics: benzimidazoles (BZ), macrocyclic lactones (ML), and imidazothiazoles including levamisole (LEV). The widespread and uncontrolled use of anthelmintics has resulted in the emergence of anthelmintic resistance (AR). In some countries the proportion of resistant GIN strains is currently so high that precludes effective control of parasitic diseases [2].

Several factors are responsible for the development of AR in parasites, of which most important are a high treatment frequency [3], underdosing of the anthelmintics, and continuous use of the same anthelmintic class over several years [4]. These factors, together with certain types of farm management, can promote the development of AR, especially in goats which need higher doses to ensure anthelmintic efficacy since they metabolise and eliminate various medicines quicker than sheep and cattle [5, 6].

Several in vivo and in vitro tests have been developed for detection of AR. An in vivo fecal egg count reduction test (FECRT) is recommended by the World Association for the Advancement of Veterinary Parasitology (WAAVP). However, this test requires animals in a herd to be either tested twice which makes it expensive, time-consuming and laborious, or a randomly selected group of animals in a herd be left untreated which, in turn, is impractical and hardly acceptable to farmers. Moreover, high inter-animal variation in the pharmacokinetics of anthelmintics in goats may lower the quality of FECRT results [5, 6]. The use of some in vitro methods like egg hatch test (EHT) or molecular tests (RT-PCR or pyrosequencing) is currently limited to BZ [7]. Moreover, EHT can be performed only on fresh fecal samples containing only eggs in early stage of development, while molecular tests are expensive and require specialized equipment in the laboratory, which makes them unsuited for routine AR diagnostics. Therefore, the most efficient in vitro test is the larval development test (LDT) which currently exists in several modifications allowing detection of AR to all three anthelmintic classes [7–10]. The LDT offers an alternative to the laborious in vivo FECRT and allows investigation of AR to all anthelmintic classes in a single test regardless of the herd size [11]. Moreover, the LDT is the only in vitro AR diagnostic test that has been commercialized and registered on the market as DrenchRite® [12].

Thus far, a number of reports have been published on the occurrence of AR in goat herds in Europe, and the prevalence of AR especially to BZ appears to be very high in some European countries (Rose et al. 2015). In France studies have shown a prevalence of AR to BZ to vary between 70 to 100% [13, 14]. Very close are results of a recent Slovakian study [15]. Many studies have demonstrated the existence of AR to the main anthelmintic classes in goat herds outside of Europe, namely in the United States [11, 16], Cuba [17], Kenya [18], South Africa [19], Uganda [20], Ethiopia [21], Malaysia [22, 23], India [24–26], and Pakistan [27].

Data on the AR situation in Polish small ruminant population are only fragmentary. In the last two decades, sporadic cases of AR to BZ in sheep, cattle, horses, pigs and goats have been reported [28–31]. Recently, first cases of AR to ML and LEV, as well as multidrug resistance (MDR) in goat herds have been described [32, 33].

On the other hand, our long cooperation with a number of Polish goat farmers has shown not only that GIN infections constitute a considerable clinical problem but also that the factors predisposing to the development of AR are present in the vast majority of goat herds. Therefore, we hypothesized that those cases of AR reported so far were only the tip of the iceberg, and we decided to carry out a large-scale epidemiological observational study to determine the prevalence of AR to the three basic anthelmintic classes in Polish goat herds and identify the genus or species of resistant larvae.

## Results

### Goat herd characteristics

Forty-two dairy goat herds were enrolled in this cross-sectional study (49% of 85 herds invited). They were evenly scattered over the entire country (located in 13 of 16 provinces of Poland). They counted from 4 to 155 adult goats with the median (IQR) of 15 (10 to 23) goats. Only 5 herds (12%) consisted of more than 50 adult goats and only 2 of them counted more than 100 heads. In most of them two Polish local breeds, Polish White Improved and Polish Fawn Improved, were kept. Nine herds (21%) kept only Anglo-Nubian goats and three herds (7%) kept a traditional local goat breed – Carpathian. In the vast majority of herds (39 of 42; 93%) goats were grazed from April to October. In 10 herds (24%) goats were grazed together on the same pasture with sheep and in 8 herds (19%) with cattle. Only 13 herds (31%) relied solely on their own replacement, the remaining 29 herds (69%) purchased goats from other herds.

Only 3 herds (7%) did not practice routine deworming. In the remaining 39 herds (93%) at least one anthelmintic was routinely used. Most of the herds which practiced routine deworming (*n* = 26; 67%) dewormed twice a year, 7 herds (18%) once a year, and 6 herds (15%) three or four times a year. BZ (albendazole and fenbendazole) were used in 36 herds (92% of herds practicing routine deworming), ML (eprinomectin, rarely ivermectin) in 33 herds (85%) and LEV in only 5 herds (13%).

### Prevalence of anthelmintic resistance

AR to each of the anthelmintic classes was detected using LDT in Polish goat herds. The most prevalent was AR to ML detected in 40 herds (95%, CI 95%: 84 to 99%) and to BZ detected in 37 herds (88%, CI 95%: 75 to 95%). AR to LEV was detected in 5 herds (12%, CI 95%: 5 to 25%) (Table 1).

**Table 1:** Results of the larval development test (LDT) in 42 Polish goat herds

Herd No.	No. of adult goats	Benzimidazoles					Macrocyclic lactones					Levamisole				
		PD_c_ (%)	cPD at the DC of 0.08 μg/ml (%) with CI 95%	EC_50_ (μg/ml)	EC_99_ (μg/ml)	AR	PD_c_ (%)	cPD at the DC of 21.6 ng/ml (%) with CI 95%	EC_50_ (ng/ml)	EC_99_ (ng/ml)	AR	PD_c_ (%)	cPD at the DC of 2.0 μg/ml (%) with CI 95%	EC_50_ (μg/ml)	EC_99_ (μg/ml)	AR
1	10	98.0	0	0.009	0.011	ND	98.0	70.0 (61.2–77.6)	36.6	546.2	severe	98.0	0	0.41	1.88	ND
2	14	98.0	99.0 (96.4–99.7)	0.273	0.793	severe	97.4	97.7 (94.1–99.1)	134.3	2374	severe	100	9.1 (4.6–17.3)	0.50	3.12	mild
3	16	95.0	42.1 (34.5–50.1)	0.082	1.054	moderate	95.0	41.0 (33.0–49.6)	20.1	924.5	moderate	95.0	0	0.55	2.04	ND
4	26	98.4	77.5 (71.4–82.6)	0.301	5.323	severe	98.4	70.1 (62.3–76.9)	49.4	4891	severe	93.2	0	0.37	1.7	ND
5	5	97.8	31.7 (25.6–38.4)	0.030	2.990	moderate	97.8	59.3 (52.4–65.9)	33.1	1524	severe	97.8	1.0 (0.3–3.6)	0.45	1.13	ND
6	8	98.4	53.9 (46.9–60.6)	0.091	1.354	severe	98.4	100 (98.0–100)	109.9	1094	severe	98.4	3.6 (1.7–7.1)	0.45	2.82	mild
7	4	100	81 (73.7–86.6)	0.165	1.645	severe	100.0	93.3 (87.7–96.4)	148.4	6831	severe	100	0	0.03	0.06	ND
8	35	98.8	97.2 (90.6–99.2)	0.333	0.924	severe	98.8	83.0 (76.2–88.1)	81.5	2169	severe	98.8	0	0.22	0.48	ND
9	40	98.6	2.2 (0.7–7)	0.007	0.042	ND	98.6	53.7 (40.3–66.7)	12.2	560.7	severe	98.6	0	0.27	0.59	ND
10	7	98.7	14.0 (9.5–20.3)	0.014	0.135	moderate	98.7	45.3 (38.4–52.4)	27.1	722.1	moderate	98.7	34.8 (24.3–47.0)	0.74	231.3	moderate
11	23	97.3	91.0 (85.2–94.7)	0.223	0.829	severe	97.3	72.3 (65.1–78.5)	121.5	257,438	severe	97.3	54.4 (40.9–67.3)	2.72	269.1	severe
12	155	98.2	7.3 (3.4–14.9)	0.030	0.300	mild	98.2	85.0 (78.6–89.7)	33.1	329.5	severe	98.2	0	0.30	0.95	ND
13	123	98.6	85.0 (78.4–89.8)	0.223	1.032	severe	98.6	82.8 (76.7–87.5)	99.5	4579	severe	98.6	0	0.45	1.13	ND
14	13	93.2	31.8 (25.1–39.3)	0.050	4.929	moderate	93.2	61.3 (54.1–68.1)	33.1	3278	severe	92.9	0	0.06	0.38	ND
15	11	95.0	84.9 (78.7–89.5)	0.247	1.991	severe	95.0	97.1 (93.5–98.7)	134.3	1725	severe	95	0	0.45	1.89	ND
16	17	86.8	27.9 (20.6–36.5)	0.050	1.065	moderate	86.8	52.4 (43.9–60.8)	24.5	7661	severe	86.8	0	0.22	1.80	ND
17	13	86.8	10.7 (7.1–15.8)	0.018	0.392	moderate	86.8	52.1 (44.9–59.3)	27.1	1248	severe	86.8	0	0.15	1.49	ND
18	10	97.9	56.3 (47.7–64.6)	0.081	3.778	severe	97.9	77.2 (69.7–83.3)	73.7	1577	severe	97.9	0	0.18	0.85	ND
19	20	98.7	0	0.003	0.008	ND	98.7	65.3 (55.0–74.3)	30.0	2966	severe	98.7	0	0.08	0.31	ND
20	5	96.2	92.2 (86.3–95.7)	0.183	0.679	severe	96.2	79.5 (72.3–85.2)	73.7	3392	severe	96.2	4.8 (2.3–9.8)	0.30	6.45	mild
21	21	100	6.3 (3.2–11.9)	0.014	0.290	mild	100	80.9 (72.3–87.3)	66.7	11,000	severe	100	0	0.14	0.63	ND
22	72	92.4	0	0.003	0.014	ND	92.4	20.0 (14.8–26.4)	9.0	240.4	moderate	92.4	0	0.10	0.63	ND
23	11	91.6	15.3 (11.0–20.9)	0.025	0.529	moderate	91.6	37.7 (31.2–44.6)	11.0	1091	moderate	91.6	1.1 (0.3–3.7)	0.09	1.17	ND
24	14	97.0	84.5 (78.9–88.9)	0.607	189.4	severe	97.0	63.9 (57.1–70.3)	54.6	5405	severe	97.0	0	0.18	1.15	ND
25	26	94.0	18.6 (13.8–24.6)	0.022	2.215	moderate	94.0	18.6 (13.8–24.6)	4.5	1400	moderate	94.0	0	0.10	0.37	ND
26	10	94.0	42.6 (35.9–49.5)	0.074	3.419	moderate	94.0	50.5 (43.7–57.4)	24.5	244.1	severe	94.0	0	0.13	0.48	ND
27	8	90.8	22.0 (16.8–28.3)	0.015	4.683	moderate	90.8	0	1.6	16.4	ND	90.8	0	0.09	0.42	ND
28	16	96.8	94.5 (90.5–96.9)	0.905	9.003	severe	96.8	52.2 (45.3–59)	22.2	285.1	severe	96.8	0	0.18	0.58	ND
29	10	93.2	91.2 (86.5–94.4)	0.741	7.371	severe	93.2	92.8 (88.4–95.6)	54.6	252.6	severe	93.2	0	0.25	0.62	ND
30	16	92.2	32.0 (25.9–38.7)	0.041	0.872	moderate	92.2	94.9 (90.9–97.2)	49.4	183.6	severe	92.6	0	0.20	0.51	ND
31	20	94.8	95.5 (91.6–97.6)	0.497	6.378	severe	94.8	96 (92.3–98)	90.0	283.9	severe	94.8	0	0.22	0.70	ND
32	9	98.8	31.8 (24.8–39.8)	0.061	0.605	moderate	98.8	93.4 (87.5–96.7)	73.7	463.1	severe	98.8	0	0.45	1.42	ND
33	76	97.9	0	0.001	0.001	ND	97.9	0	0.1	13.4	ND	97.9	0	0.06	0.38	ND
34	13	98.9	91.4 (86.0–94.8)	0.497	155.1	severe	98.9	67.1 (59.8–73.7)	54.6	5405	severe	98.9	0	0.18	0.74	ND
35	10	97.8	92.6 (86.0–96.2)	0.368	7.873	severe	97.8	96.3 (91–98.5)	73.7	595.1	severe	97.8	0	0.27	0.72	ND
36	27	90.6	81.7 (75.7–86.4)	0.368	9.798	severe	90.6	79.4 (72.4–85)	44.7	956.6	severe	90.6	0	0.20	0.93	ND
37	5	96.8	51.5 (44.4–58.5)	0.082	13.54	severe	96.8	5.3 (2.5–11.0)	3.7	47.1	mild	96.8	0	0.20	0.64	ND
38	6	97.0	90.7 (84.1–94.8)	1.649	974.7	severe	97.0	83.5 (75.6–89.2)	54.6	543.2	severe	97.0	0	0.27	0.86	ND
39	73	97.2	88.0 (81.0–92.6)	0.301	5.232	severe	97.2	92.0 (85.9–95.6)	109.9	2353	severe	97.2	0.9 (0.2–4.5)	0.47	2.19	ND
40	22	88.9	57.9 (50.6–64.9)	0.135	13.40	severe	88.9	7.9 (4.8–12.7)	9.0	56.7	mild	88.9	0	0.17	0.41	ND
41	21	98.8	63.4 (54.8–71.2)	0.165	16.37	severe	98.8	94.9 (90.4–97.4)	121.5	562.1	severe	98.8	0	0.18	0.39	ND
42	18	93.2	63.3 (53.5–72.1)	0.150	3.201	severe	93.2	29.0 (21.0–38.5)	12.2	260.7	moderate	93.2	0	0.33	1.05	ND

*AR* anthelmintic resistance, *DC* discriminating concentration, *PD*_*c*_ percentage of developing larvae in the control wells, *cPD at the DC* corrected percentage of larvae developing in tested wells at the discriminating concentration of a given anthelmintic agent, *CI 95* 95% confidence interval, *EC*_*50*_ median effective concentration, *EC*_*99*_ the 99th percentile effective concentration, *ND* not detected

**Fig. 1:**
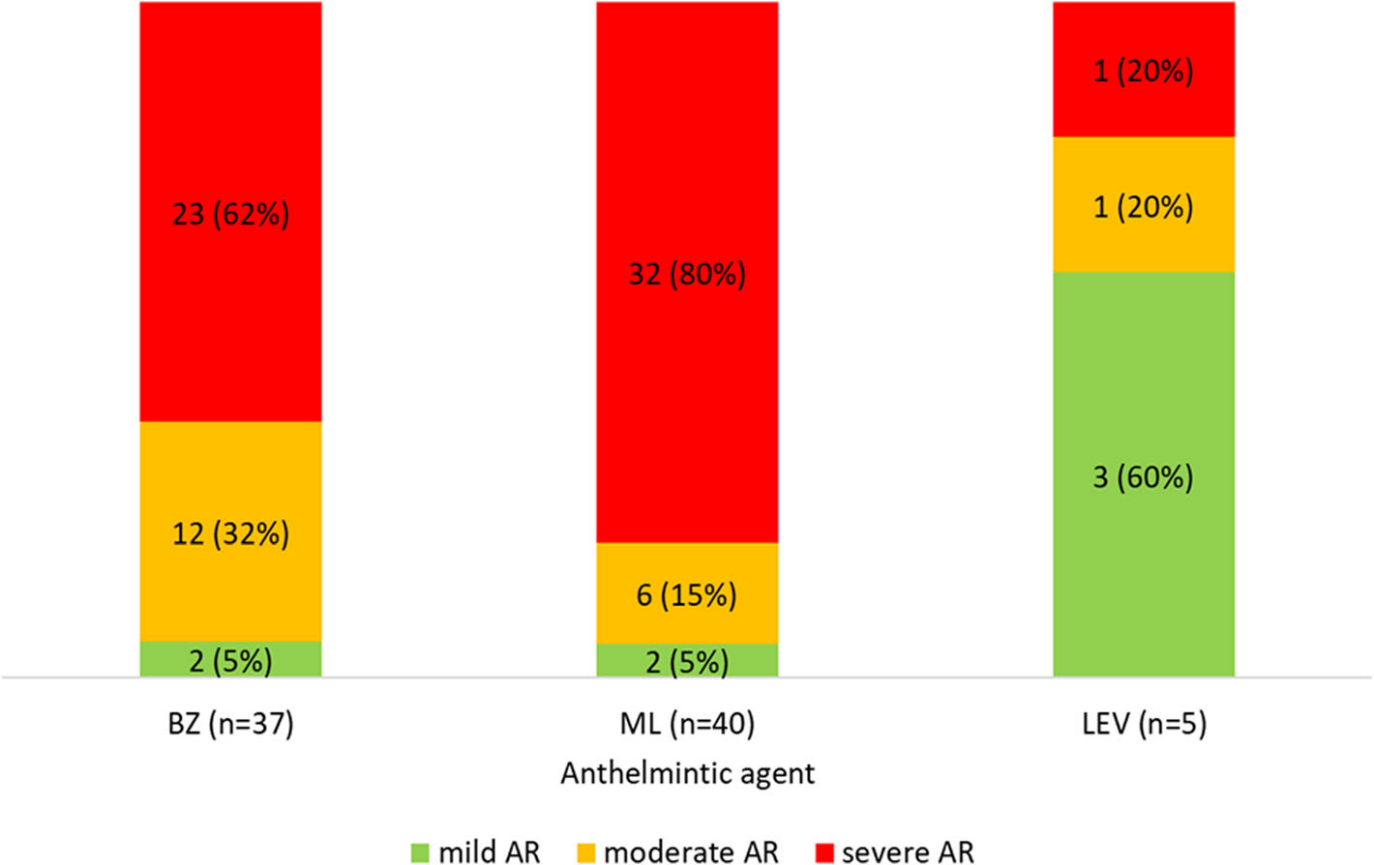
The number (percentage) of herds harbouring resistant GIN populations of various severity of anthelmintic resistance (AR) to benzimidazoles (BZ), macrocyclic lactones (ML) and levamisole (LEV) in Polish goat population classed as mild (< 10% of larvae developed at the discriminating concentration), moderate (10–50% of larvae developed), and severe (> 50% of larvae developed)

Only one herd was free from AR to any of anthelmintics. AR to one anthelmintic was found in 5 herds (AR to ML in 4 herds, and to BZ in one herd), AR to two anthelmintics in 31 herds (BZ and ML in all) and AR to all three anthelmintics (synonymous to MDR) in 5 herds, which yielded the prevalence of MDR to be 12% (CI 95%: 5 to 25%). In most of herds AR to BZ and ML was severe (> 50% of larvae developed at the discriminating concentration, DC), while AR to LEV was mostly mild (< 10% of larvae developed at the DC) (Fig. 1).

Corrected percentage of larvae developing in tested wells (cPD) at the DC was significantly positively correlated with EC_50_ for all three anthelmintic classes and this correlation was high for BZ (R_s_ = 0.94, *p* < 0.001) and ML (R_s_ = 0.90, p < 0.001), and moderate for LEV (R_s_ = 0.48, *p* = 0.001) (Fig. S1). EC_50_ and EC_99_ for susceptible and resistant GIN populations are summarized in Table 2.

**Table 2:** Effective concentrations of anthelmintics inhibiting 50% (EC_50_) and 99% (EC_99_) of larvae in gastrointestinal nematode (GIN) populations from herds classified as susceptible and resistant, presented as the median, interquartile range (IQR), and range in parenthesis

GIN population	No. of herds	EC_50_	EC_99_
**Benzimidazoles – discriminating concentration (DC) = 0.08 μg/ml**			
susceptible	5	0.003, 0.003–0.007 (0.001–0.009)	0.011, 0.008–0.014 (0.001–0.042)
resistant	37	0.150, 0.050–0.301 (0.014–1.649)	2.990, 0.872–7.371 (0.135–974.682)
**Macrocyclic lactones – DC = 21.6 ng/ml**			
susceptible	2	0.892 (0.135–1.649)	14.902 (13.398–16.400)
resistant	40	49.402, 24.533–77.576 (3.669–148.413)	1092.628, 396.322–3122.438 (47.127–257,437.7)
**Levamisole – DC = 2.0** μg**/ml**			
susceptible	37	0.202, 0.135–0.301 (0.025–0.549)	0.724, 0.480–1.148 (0.062–2.185^a^)
resistant	5	0.497, 0.449–0.741 (0.301–2.718)	6.446, 3.121–231.343 (2.824–269.110)

^a^ GIN populations from two herds (no. 5 and 23 in Table 1) were classified as susceptible despite ED_99_ > 2 μg/ml because the corrected percentage larval development at the DC was ≤1%

cPD at the DC in BZ-resistant populations ranged from 6 to 99% with the median (IQR) of 63% (32 to 91%), and was not significantly different from cPD at the DC in ML-resistant populations which ranged from 5 to 100% with the median (IQR) of 71% (52 to 92%) (*p* = 0.883). cPD at the DC in LEV-resistant populations ranged from 4 to 54% with the median of 9%, and was significantly lower than both the former (*p* = 0.047) and the latter GIN populations (*p* = 0.010).

### Gastrointestinal nematode populations

*H. contortus* was present in 41 herds (98%, CI 95%: 88 to 100%), *Trichostrongylus* spp. in 37 herds (88%, CI 95%: 75 to 95%), *Oesophagostomum* spp. in 22 herds (52%, CI 95%: 38 to 67%), and *Teladorsagia* spp. in 14 herds (33%, CI 95%: 21 to 48%). Median fecal egg count (FEC) in the herds ranged from 75 to 2450 epg.

**Fig. 2:**
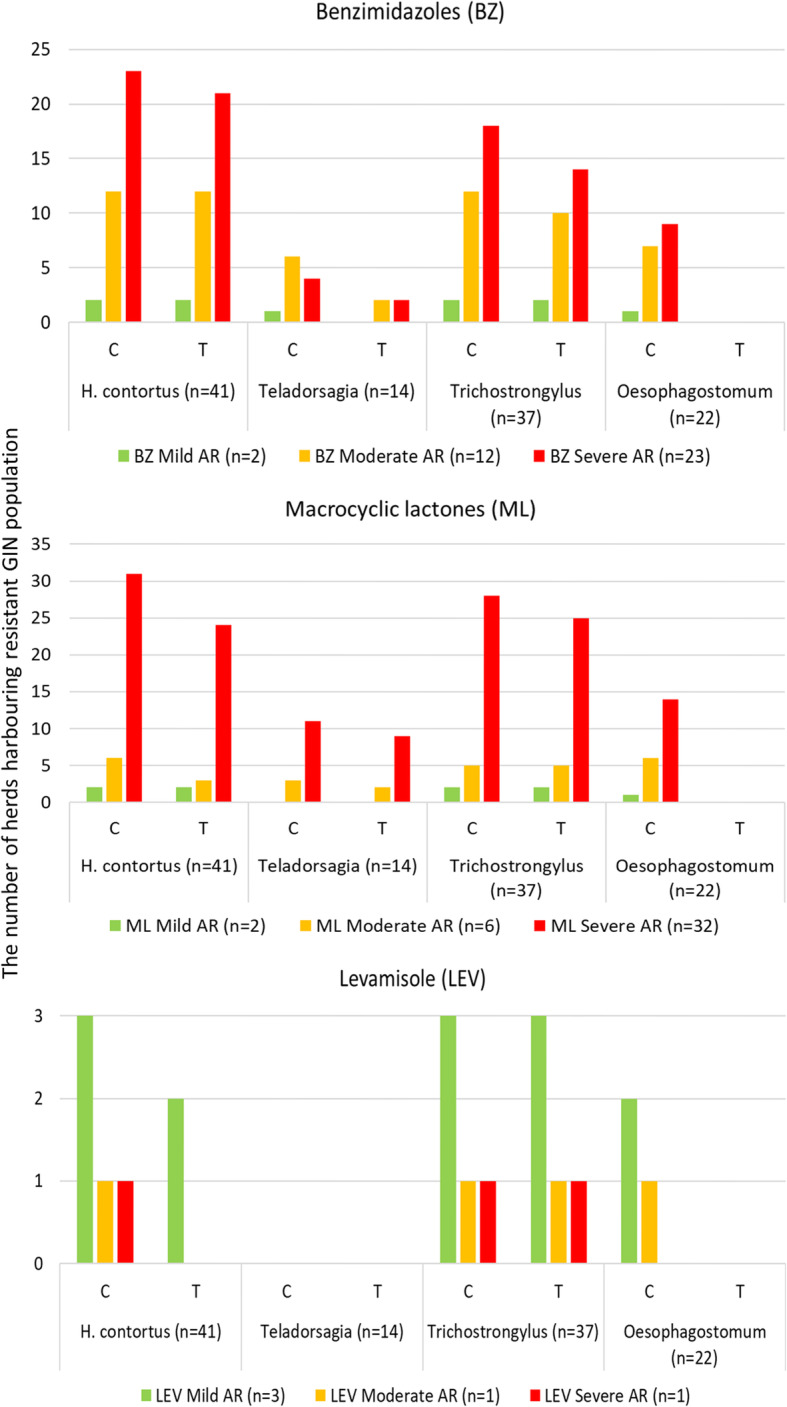
The number of herds harbouring GIN populations resistant to benzimidazoles (BZ), macrocyclic lactones (ML), and levamisole (LEV) in which larvae developed in control wells (C) and in tested wells (T) at the discriminating concentration (DC). Anthelmintic resistance was classed as mild (< 10% of larvae developed at the DC), moderate (10–50% of larvae), and severe (> 50% of larvae). Letter “n” stands for the number of herds infected with a relevant GIN

At the DC, no *Oesophagostomum* larvae developed irrespective of the herd’s AR status. In the case of BZ-resistance *H. contortus* larvae developed at the DC in 95% of the populations which initially harboured this GIN, *Trichostrongylus* larvae in 81% of the populations, and *Teladorsagia* larvae in 36% of the populations. In the case of ML-resistance *Trichostrongylus* larvae developed at the DC in 91% of the populations, *Teladorsagia* larvae in 79% of the populations and *H. contortus* larvae in 74% of the populations. In the case of LEV-resistance *Trichostrongylus* larvae developed at the DC in 100% of the populations, while *H. contortus* larvae in 20% of the populations, and *Teladorsagia* larvae in no population. Of 5 herds resistant to LEV, 3 had *Trichostrongylus* spp. as the only GIN left in wells with LEV at the DC, and 2 had *Trichostrongylus* spp. and *H. contortus*. These results indicated that *H. contortus* spp. and *Trichostrongylus* spp. were mainly responsible for AR to BZ, all three GIN were responsible for AR to ML, while *Trichostrongylus* spp. was the main GIN resistant to LEV (Fig. 2).

### Factors linked to AR

Presence of AR to BZ and ML in virtually all investigated herds reduced the reliability of most statistical analyses. The number of times goats had been dewormed was significantly positively linked with the intensity of AR to BZ (*p* = 0.007), but not to ML (*p* = 0.643) or LEV (*p* = 0.849). The use of BZ was significantly related to the presence of AR to BZ (*p* = 0.002), however there was no significant relationship between the use of ML and LEV and the presence of relevant AR (*p* = 0.449 and *p* = 0.099, respectively). Interestingly, AR to LEV was observed in 3 herds whose owners declared not to have used LEV in previous 3 years. The same applied to 9 herds with AR to ML, and 3 herds with AR to BZ. None of the following: access to pasture, introducing goats from other herds, and grazing with sheep or cattle on common pasture was significantly linked with AR to any of the anthelmintics.

## Discussion

Our cross-sectional study shows that AR to BZ and ML is widespread in Polish goat herds, whereas AR to LEV is uncommon. This is the first study systematically investigating the prevalence of AR to three basic classes of anthelmintics in small ruminants in Poland. Similar surveys based on in vitro tests have so far been conducted in Europe on sheep flocks in Lithuania [9, 34] and Ireland [35], and sheep and goat flocks in Slovakia [15, 36]. Moreover, the LDT is a commonly applied technique in the AR prevalence studies in the United States [11, 16, 37, 38], Philippines [39, 40], Canada [41] and the New Zealand [42, 43] in both goat and sheep flocks. The LDT allows to estimate efficacy of anthelmintics in nematode populations from small farms where performance of a FECRT for even one anthelmintic would be impractical [11, 40]. This is the case in Poland, where most of goat herds consist of only a few animals.

Anthelmintics most often used in goats in Poland are albendazole, fenbendazole and eprinomectin, while levamisole is used rarely and this fact appears to directly correspond to the prevalence of AR. BZ are relatively inexpensive and have short withdrawal periods, which matters a lot to Polish farmers as their herds mainly produce milk and cheese. Moreover, BZ are believed to eliminate tapeworms and liver flukes which are commonly considered by farmers widespread and dangerous for goats, even though our observations seem to contradict those statements [44]. Anyway, several decades of relying virtually solely on BZ must have led to the point at which we are now – the vast majority of herds harbour resistant GIN. Cold comfort is the fact that Polish situation does not seem to be much distinct from other European countries where prevalence of AR to BZ in goats is equally high [3, 15, 45].

Surprisingly, AR to ML is equally widespread in Poland, yet the first case was described only a year ago [32]. The prevalence of AR to ML in goats varies between European countries from 0% in Norway [46], 20% in Italy [47] to 100% in some regions of Switzerland [48]. Situation in Poland is closer to this in Switzerland and AR to ML in goats has already exceeded 90%. The most important cause seems to be the presence on the Polish veterinary market of eprinomectin in pour-on formulation for cattle with milk withdrawal period of 0 days (routinely extrapolated to goats). Pour-on formulation makes its use very simple and eprinomectin is also commonly used in Poland to control external parasite invasions without taking into account its simultaneous effect on GIN. These facts make it one of the most popular anthelmintic agents in Poland. However, eprinomectin is very likely to be commonly underdosed in Polish goat population since the appropriate dose of eprinomectin against GIN in goats is at least two times higher than that for cattle [49, 50], Unfortunately, our experience shows that this fact is unknown or routinely neglected by farmers and veterinarians, also for financial reasons. As all avermectins have the same mechanism of action the cross-resistance with ivermectin is complete [51]. Moreover, it has been suggested that ML may select single nucleotide polymorphism at BZ-resistance-associated codons of β-tubulin [52, 53]. As a consequence, AR to ML and BZ appear to be mutually related phenomena and frequent use of ML may stimulate the development of BZ resistance.

AR to LEV in Polish goat population is still uncommon and mild, which appears to result from its rare use. LEV is viewed by veterinarians as an old-fashioned drug with narrow margin of safety and low effectiveness so they prefer to avoid it. Data on the prevalence of AR to LEV in goats in Europe are limited, mainly case reports have been published in Denmark [54], France [13, 14], and the UK [55]. Recent, large-scale Dutch study did not reveal AR to LEV in sheep flocks [56].

Interestingly, AR to certain anthelmintic classes was observed on some farms where their owners declared not to have used this particular anthelmintic class at all. We suppose there are three possible explanations to this phenomenon, none of which excludes the others. First, the farmers may have not been fully aware of anthelmintics used since some of them are administered by veterinarians during consultation on the farm, especially in the case of injectable anthelmintics. Secondly, our questionnaire applied to quite a long period of time (3 years) and it is likely that farmers could have simply forgotten the facts of using of anthelmintics on their farms. And thirdly, there could be some goats purchased from other herds in Poland or abroad which had been dewormed with this particular anthelmintic and thus carried resistant GIN population. It is mainly the case in herds with AR to LEV and without previous use of this anthelmintic as in all these herds Anglo-Nubian goats were kept and goats of this breed are usually imported to Poland from abroad, mostly from Germany where AR to all three anthelmintic classes in small ruminants has so far been detected [57]. Moreover, owners of these three herds declared in the questionnaire to have purchased goats in the past.

Multidrug resistance of GIN in goats in Europe is not widespread [45], and only a few cases have so far been reported in Denmark [54], France [13], Switzerland [58], UK [59] and Poland [33]. Given that on most of farms BZ and ML are used, either separately, simultaneously or interchangeably, multidrug AR will most likely develop in those herds which, seeking the solution for dropping effectiveness of routine deworming, will switch to levamisole and use it intensively for some time. As AR to LEV seems to develop quite quickly [60] a year of routine LEV use suffices to produce AR.

In our study we decided to use the DC rather than the EC_50_ criterion for differentiation between resistant and susceptible GIN as an application of the EC_50_ may sometimes underestimate the resistance and thus some herds might have been incorrectly classed by this criterion as susceptible [61]. It has been suggested that using the EC_99_ values or the DC in the in vitro tests such as the LDT or EHT can substantially increase test sensitivity of the test and identify resistance when only a small proportion of the GIN population is resistant [8, 61, 62]. To maintain high accuracy of our classification we decided to use two criteria, one based on the corrected percentage of development at the DC and the another based on EC_99_, simultaneously.

AR to BZ and ML seems to be conveyed by three main GIN which we identified in our study – *H. contortus*, *Trichostrongylus* spp., and *Teladorsagia* spp. Only *Oesophagostomum* spp. showed no potential to become resistant to any anthelmintic which is at odds with some recent studies in which *Oesophagostomum* spp. was together with *H. contortus* and *Trichostrongylus* spp. resistant to BZ and ML [63, 64]. Moreover, our observation has to be treated with caution as the only DC which has been scientifically proven to indicate AR is the DC of IVM-AG for *H. contortus*. Initially, it had been set at 5.4 ng/ml, however then its elevation to 21.6 ng/ml was suggested [8], and as a consequence we decided to use the higher DC as a safer option. However, nothing is known about the DC indicating AR in *Trichostrongylus* spp. or *Teladorsagia* sp. so we cannot be sure that the development of these GIN at this DC corresponds to their resistance to ML. Even though it is very likely to be so, it undoubtedly warrants further investigation. Interestingly, it seems that *Trichostrongylus* spp., not *H. contortus* as we initially expected, is the main culprit responsible for the early stage of development of AR to LEV. We made this observation first in our recent study [33] and this survey appears to confirm it, however we do not know any studies regarding this topic in goats. Studies so far conducted in sheep and goats have indicated that both *Trichostrongylus* spp. and *H. contortus* are responsible for AR to LEV [11, 13, 14, 16, 25, 37, 38, 54, 57, 63–69].

Our work presents the results of an in vitro test (LDT) which may in some circumstances differ from those obtained in an in vivo method such as FECRT. Such discrepancies have multifactorial background and most of these factors remain beyond researcher’s control [15, 70, 71]. Nevertheless, LDT and FECRT have been proven to show moderate to good agreement, especially regarding BZ and LEV resistance detection [10, 15, 72, 73]. Therefore, our results are likely to present the true AR status of the examined goat herds.

The main drawback to our study is the fact that herds enrolled in the survey were not randomly selected. Their owners had sent fecal samples to our laboratory at least once before so they were probably somehow aware of the importance of parasite control in goats. Given that most of them used to deworm all their goats routinely, usually using the same drug for a long time, they were more likely to promote the development of AR on their farms. Therefore, it is possible that our results may to some extent overestimate AR prevalence in the country, and we hope they do so. Anyway, they allow to realize that this problem already exists in Poland and unless actions are undertaken to stop it, soon no anthelmintic will remain effective in some goat herds. The another drawback to our study is that when we performed LDT with LEV we wrongly used 1.3% DMSO in control wells while LEV was diluted in water (contrary to TBZ and IVM-AG which were diluted in 1.3% DMSO). As a result if 1.3% DMSO had a potential to reduce larval development, this mistake could have falsely increased the corrected percentage of developing larvae in tested wells at the DC. Our previous observations have not indicated that DMSO at such a low concentration impacts on larval development to any noteworthy extent (unpublished data). However, even if it did we hope that the double criterion we used for classification GIN populations as resistant would reduce the risk of falsely positive result to an acceptable minimum.

## Conclusions

This study provides the first comprehensive data on the prevalence of AR in goat herds in Poland. AR to BZ and ML is widespread, while AR to LEV is currently at a low level. Therefore, an appropriate strategy of GIN control should be applied by farmers and veterinarians as soon as possible not to let the situation slip out of control.

## Methods

### Study design

This was a cross-sectional study and lasted virtually one year, from September 2018 to June 2019. During this time the owners of 85 private dairy goat herds who had cooperated with our laboratory in previous 4 years (2015–2018), were invited to voluntarily enrol in the study. Those who declared participation were asked to collect fecal samples after at least 8 weeks since the last deworming had passed, and detailed instructions on proper collection and preparation of anaerobically stored fecal samples were sent to them. In herds which consisted of less than 15 adult goats fecal samples were collected from all animals, otherwise samples were collected from 15 to 20 goats selected by the owners. The owners were asked to pick only these goats which had been present on their farm for at least 8 previous weeks to avoid including goats dewormed within this period of time by the former owner. Fecal samples were collected by the farmers (owners of the goats) directly from the rectum. Pooled fecal samples weighing 50–100 g were placed in plastic containers filled with tap water to ensure anaerobic conditions. Samples were delivered to the laboratory within 24 h at room temperature and processed within next 24 h so that the total time which had elapsed between sample collection and examination did not exceed 48 h.

In each herd the following information was collected using the questionnaire: the number of adult goats (> 6 month-old) and kids, access to the pasture, co-grazing or rotational grazing with sheep and cattle, purchase of goats from other herds, and the history of deworming including the number of times the herd had been dewormed in previous 3 years and drugs used.

According to Polish legal regulations (The Act of the Polish Parliament of 15 January 2015 on the Protection of Animals Used for Scientific or Educational Purposes, Journal of Laws 2015, item 266) no formal ethics consent was required for this study except for the informed consent of participants, which we obtained in written from each participating farmer.

### Larval development test

Based on the technique defined by Hubert and Kerboeuf [74], the larval development test (LDT) was performed with the modifications of Várady et al. [75, 76]. Nematode eggs were collected by sequentially sieving the anaerobically stored fecal samples through stacked sieves with apertures of 250, 100 and 25 μm. The material collected on the 25 μm sieve was washed with tap water, sedimented, and then flotation method with saturated sodium chloride was applied in order to obtain the suspension of the nematodes eggs [7]. After extraction, eggs were inspected microscopically to ensure that embryonation had not yet begun and suspended in deionized water at a concentration of 70–100 eggs in 10 μl per well. The pure thiabendazole (Sigma-Aldrich, Merck, Germany; TBZ), ivermectin aglycone (Santa Cruz Biotechnology, USA; IVM-AG), and levamisole (Sigma-Aldrich, Merck, Germany; LEV) were firstly dissolved in ≥99.5% dimethyl sulfoxide (Sigma-Aldrich, Merck, Germany; DMSO) in case of TBZ and IVM-AG or deionized water in case of LEV and serially diluted 1:2 in DMSO (TBZ, IVM-AG) or in deionized water (LEV). The overall DMSO concentration in the pre-dilution plate was 20%. Then, diluted anthelmintics and DMSO (control) were moved to the test plate to yield final concentration of 1.3% in the tested wells. The 12 concentrations of TBZ, IVM-AG and LEV finally used in the LDT ranged from 0.0006 to 1.28 μg/ml, from 0.084 to 173.6 ng/ml, and from 0.020 to 32 μg/ml, respectively. Test was performed on 96-well cell culture plates (Sarsted, Germany) with culture medium (150 μl) which consisted of 10 μl of (all in one test plate) TBZ, IVM-AG, LEV or DMSO (1.3%; control wells) solution, 110 μl of deionised water, 20 μl of culture medium as described by Hubert and Kerboeuf [77] and 10 μl of a suspension (approximately 70–100 eggs) containing Amphotericin B (Sigma-Aldrich, Merck, Germany) at a concentration of 5 μg/ml. Each anthelmintic concentration was tested in duplicate. The LDT plates (sealed – to prevent drying) were incubated for 7 days at 25 °C (Cooled incubator, INCU-Line® Standard, VWR). After the incubation period, 10 μl of Lugol’s solution was added to each well to terminate development of larvae. The unhatched eggs and L1-L3 larvae were counted under an inverted microscope in each well (Olympus, CKX53, Poland) and the L3 larvae were classified at species/genus level in the tested and control wells following the procedure detailed elsewhere [78]. The arithmetic mean of the percentage of larvae developing in two tested wells at each anthelmintic concentration (percentage development in tested wells, PD_T_) was corrected by the percentage of developing larvae in the control wells (PD_C_) according to the following formula:

cPD = PD_T_ / PD_C,_

where cPD stood for corrected percentage of larvae developing in tested wells.

The concentration of each anthelmintic inhibiting development of 50% (median effective concentration, EC_50_) and 99% (EC_99_) of larvae was estimated using the 4 parameter logistic curve [79].

The results of LDT were interpreted with respect to the discriminating concentration (DC) of anthelmintic agent which was defined as the concentration of anthelmintic at which the development of at least 99% of susceptible larvae (corrected by the larvae developing in control wells as mentioned above) would have been inhibited [7, 62]. The following DC were used in the study: 0.08 μg/ml for TBZ [15], 21.6 ng/ml [34, 36] for IVM-AG, and 2.0 μg/ml for LEV [80].

Two criteria had to be simultaneously fulfilled to classify a GIN population from a given goat herd as resistant to the particular anthelmintic: cPD at the DC significantly higher than 1% (meaning that the entire 95% confidence interval (CI 95%) was above 1%) and EC_99_ > DC.

AR was arbitrarily classed in terms of severity as follows: cPD at the DC from > 1 to 10% – mild AR, from > 10 to 50% – moderate AR, above 50% – severe AR. Multidrug AR (MDR) was defined as the presence of AR to all three different classes of anthelmintics.

### Statistical analysis

Numerical variables were presented as the median, interquartile range (IQR) and range, and compared between groups using the Mann-Whitney U test (unpaired groups) or Wilcoxon signed rank test (paired groups). Categorical variables were given as count and percentage in a group, and compared between groups using the Pearson’s chi-square test or the Fisher exact test. The 95% confidence intervals (CI 95%) for percentages were calculated using the Wilson score method [81]. Correlations between cPD at the DC and EC_50_ were assessed using the Spearman’s rank correlation coefficient (R_s_). The significance level (α) was set at 0.05 and the Bonferroni correction was applied in the case of multiple comparisons. Statistical analysis was performed in TIBCO Statistica 13.3.0 (TIBCO Software Inc., Palo Alto, CA).

## Supplementary Information

**Additional file 1: Fig. S1.** Correlations (presented as the Spearman’s rank correlation coefficients, R_s_) between the corrected percentage of larvae developing in tested wells (cPD) at the discriminating concentration (DC) of each anthelmintic agent and the median effective concentration (ED_50_) of each anthelmintic agent. Scatter plots presenting correlations between cPD at DC and ED_50_ for each of anthelmintic agents.

## Data Availability

The data sets used and/or analyzed are available from the corresponding author on reasonable request.

## Abbreviations

α: Significance level
AR: Anthelmintic resistance
BZ: Benzimidazoles
CI 95%: Confidence interval for the level of confidence of 95%
cPD: Corrected percentage of developing larvae
DC: Discriminating concentration
DMSO: Dimethyl sulfoxide
EC_50_: Median effective concentration
EC_99_: The 99th percentile effective concentration
EHT: Egg hatch test
epg: Eggs per gram
FEC: Fecal egg count
FECRT: Fecal egg count reduction test
GIN: Gastrointestinal nematodes
IQR: Interquartile range
IVM: Ivermectin
IVM-AG: Ivermectin aglycone
LDT: Larval development test
LEV: Levamisole
MDR: Multidrug resistance
ML: Macrocyclic lactones
PD_C_: Percentage development in control wells
PD_T_: Percentage development in tested wells
R_s_: Spearman’s rank correlation coefficient
TBZ: Thiabendazole
