# Supplementary figures and images for: Prevalence of anthelmintic resistance of gastrointestinal nematodes in Polish goat herds assessed by the larval development test
Source: BMC Vet Res. 2021 Jan 7;17:19. doi: 10.1186/s12917-020-02721-9 (PMC7791792; doi:10.1186/s12917-020-02721-9)

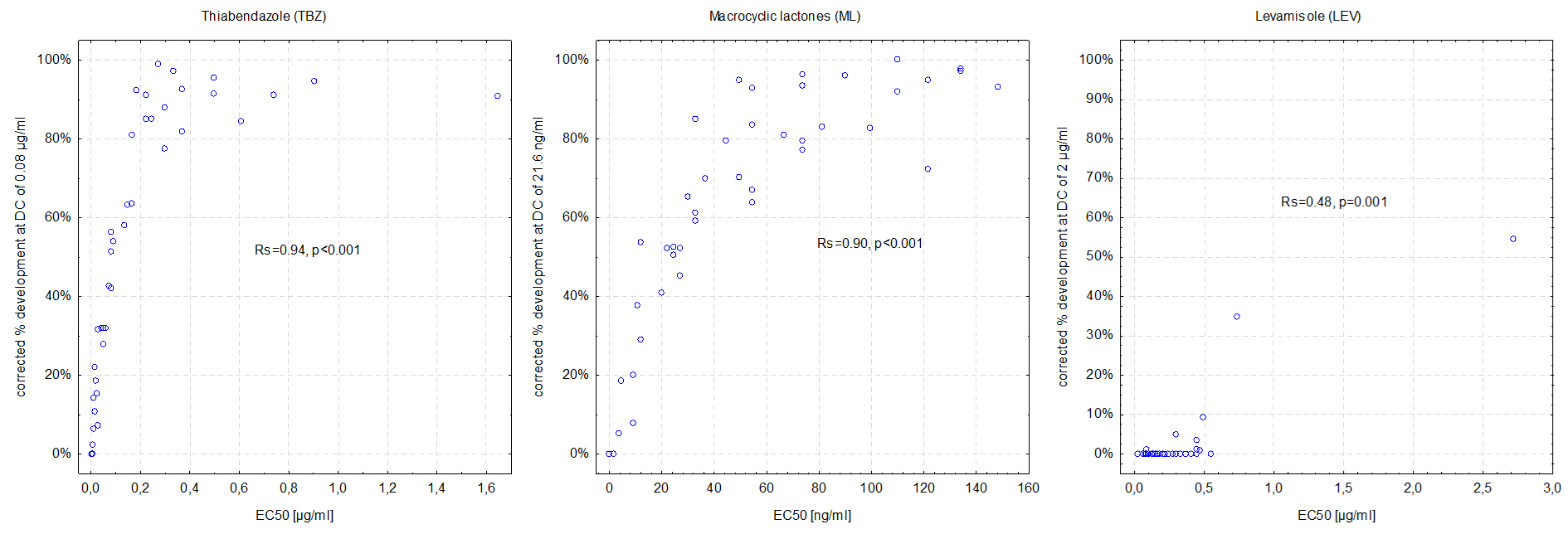

Supplement: Supplementary file 1 — Additional file 1: Fig. S1. Correlations (presented as the Spearman’s rank correlation coefficients, Rs) between the corrected percentage of larvae developing in tested wells (cPD) at the discriminating concentration (DC) of each anthelmintic agent and the median effective concentration (ED50) of each anthelmintic agent. Scatter plots presenting correlations between cPD at DC and ED50 for each of anthelmintic agents. [file 12917_2020_2721_MOESM1_ESM.tif]
